# Effects of a High-Intensity Exercise Program on Weight Regain and Cardio-metabolic Profile after 3 Years of Bariatric Surgery: A Randomized Trial

**DOI:** 10.1038/s41598-020-60044-z

**Published:** 2020-02-20

**Authors:** A. Marc-Hernández, J. Ruiz-Tovar, A. Aracil, S. Guillén, M. Moya-Ramón

**Affiliations:** 1 Laboratory of Training Analysis and Optimization, Sport Research Center, Miguel Hernandez University, Elche, 03202 Spain; 2 Centre of Excellence for the Diagnosis and Treatment of Obesity and Diabetes, Valladolid, 47004 Spain; 3 Department of Sport Sciences, Miguel Hernandez University, Elche, 03202 Spain; 4 Instituto de Neurociencias, UMH-CSIC, Sant Joan d’Alacant, 03550 Spain; 5 Institute for Health and Biomedical Research (ISABIAL-FISABIO Foundation), Alicante, 03010 Spain

**Keywords:** Metabolic syndrome, Obesity

## Abstract

Weight regain is one of the most common problems in the long-term after bariatric surgery. It is unknown if high-intensity exercise programs applied in late phases of post-surgical follow-up could counteract this trend. After a 3-year follow-up, 21 patients underwent sleeve gastrectomy were randomized into an exercise group (EG, n = 11), that performed a 5-month supervised exercise program, and a control group (CG, n = 10), that followed the usual care. Body composition, cardiorespiratory fitness, glycaemia and blood cholesterol were evaluated before and after the intervention. Finally, the EG repeated the evaluations 2 months after the end of the exercise program. Both groups reached their maximum weight loss at the first year after surgery and showed significant weight regain by the end of the follow-up. After the exercise program, the EG showed reductions in fat mass (−2.5 ± 2.6  kg, *P* < 0.05), glycaemia (−13.4 ± 8.7  mg·dL^−1,^ *P* < 0.01) and blood cholesterol (−24.6 ± 29.1  mg·dL^−1^, *P* < 0.05), whereas the CG during the same period showed increases in weight (1.5 ± 1.3  kg, *P* < 0.05) and fat mass (1.8 ± 0.9, *P* < 0.01). Two months after the end of the program, EG had increases in weight (1.1 ± 1.2  kg, *P* < 0.05), fat mass (2.6 ± 2.2  kg, *P* < 0.01), glycaemia (8.2 ± 11.6  mg·dL^−1^, *P* < 0.05) and blood cholesterol (20.0 ± 22.1  mg·dL^−1^, *P* < 0.05), when compared with the values after the exercise program. Therefore, in the medium-term after sleeve gastrectomy exercise may contribute to prevent weight regain and to reduce fat mass, glycaemia, and blood cholesterol.

## Introduction

Bariatric surgery (BS) is considered successful when patients reach 50% or more of their excess weight loss (EWL) and achieve a body mass index (BMI) below 35  kg·m^−2^^1^. In the short-term follow-up, BS has been shown to be very effective in generating weight loss and improvements in obesity-related comorbidities and cardio-metabolic risk factors^2^. However, in the long-term (≥5 years) follow-up studies, a weight regain phase has been described for some bariatric techniques, being one of the greatest problems mainly after restrictive procedures^3^. Several studies have reported up to 58.5% of weight regain in patients undergoing sleeve gastrectomy^4–6^. As a consequence, the percentage of excess weight loss (%EWL) decreases over time^7^ and after 5 or more years after surgery, between 32% and 64% of patients are below 50% of EWL^8,9^. This weight regain, besides leading in some cases to a revisional surgery (15.4–36% of patients)^5,6^, may also be accompanied by an increase in comorbidities^10^.

Several reasons have been suggested to explain weight regain after sleeve gastrectomy, such as a greater residual gastric volume^11^, or maladaptive eating behaviours^4,12,13^. However, physical activity seems to play a relevant role in this regard. After BS, physical activity levels increase^13,14^, which is associated with increases in %EWL^15^. However, over time, physical activity levels tend to decrease^16^, which can contribute to weight regain. It has been demonstrated that bariatric patients who performed some physical activity had a lower incidence of weight regain than sedentary subjects^4^. Few studies, mostly observational, have analysed the role of physical activity in weight regain, reporting associations between a sedentary lifestyle and weight regain^4^. Similarly, in the long-term, the effects of exercise on cardio-metabolic risk factors and on the quality of life still remain unclear.

Therefore, the aim of the present study was to analyse the effects of a supervised and customized exercise program that combined endurance and resistance training, initiated at moderate intensity and progressing to high intensities applied 3 years after sleeve gastrectomy. The primary outcome was the effect on weight regain and body composition. Additional secondary outcomes were the effects on physical fitness, cardiovascular risk (CVR) and quality of life.

## Results

### Participants

**Figure 1:**
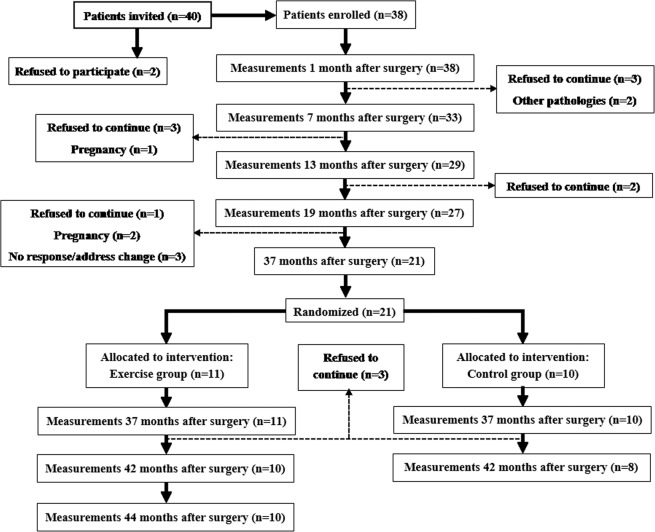
CONSORT flow diagram during the study. Two participants from the control group were lost, and one participant in the experimental group failed to complete the program due to a lesion unrelated to the intervention.

Two patients declined to participate in the study after initial interview. During the follow-up, 19 patients were lost, and 21 were enrolled at the intervention. Of these patients, 11 patients were allocated in the exercise group (EG; 72.7% women) and 10 patients were allocated in the control group (CG; 80% women). Data from two of the patients from the CG that were lost during the follow-up, and from one of the patients that failed to complete ≥85% of the intervention were excluded of the statistical analyses (Fig. 1). No significant differences between both groups were noticed at baseline (Table 1).

**Table 1:** Baseline characteristics of participants that completed the study.

	Baseline			At month 37 postoperatively		
	Total (n = 18)	CG (n = 8)	EG (n = 10)	Total (n = 18)	CG (n = 8)	EG (n = 10)
Age (years)	45.7 ± 8.9	43.7 ± 11.4	47.3 ± 6.5	48.7 ± 8.9	46.4 ± 11.2	50.6 ± 6.6
Weight (kg)	101.9 ± 18.8	98.4 ± 18.0	104.7 ± 19.8	89.0 ± 19.5	84.6 ± 15.9	92.5 ± 22.2
BMI (kg·m-²)	38.6 ± 4.8	38.2 ± 5.1	38.9 ± 4.8	33.7 ± 5.9	32.8 ± 4.3	34.4 ± 7.0
Fat mass (%)	43.9 ± 5.9	43.5 ± 5.8	44.2 ± 6.2	38.5 ± 8.9	37.5 ± 8.8	39.4 ± 9.3
Fat mass (kg)	45.1 ± 12.1	42.9 ± 9.5	46.9 ± 14.0	35.3 ± 14.6	32.3 ± 10.8	37.7 ± 17.1
Fat free mass (%)	56.1 ± 5.9	56.5 ± 5.8	55.8 ± 6.2	61.5 ± 8.9	62.5 ± 8.8	60.6 ± 9.3
Fat free mass (kg)	56.8 ± 10.1	55.5 ± 12.1	57.7 ± 8.7	53.7 ± 9.5	52.3 ± 9.8	54.8 ± 9.7
Female (%)	77.8	87.5	70.0	77.8	87.5	70.0

Data at the beginning of the follow-up (1 month after surgery), and at the start of the exercise program (37 months after surgery). CG, control group; EG, exercise group; BMI, body mass index.

### Body composition and anthropometric measures

**Figure 2:**
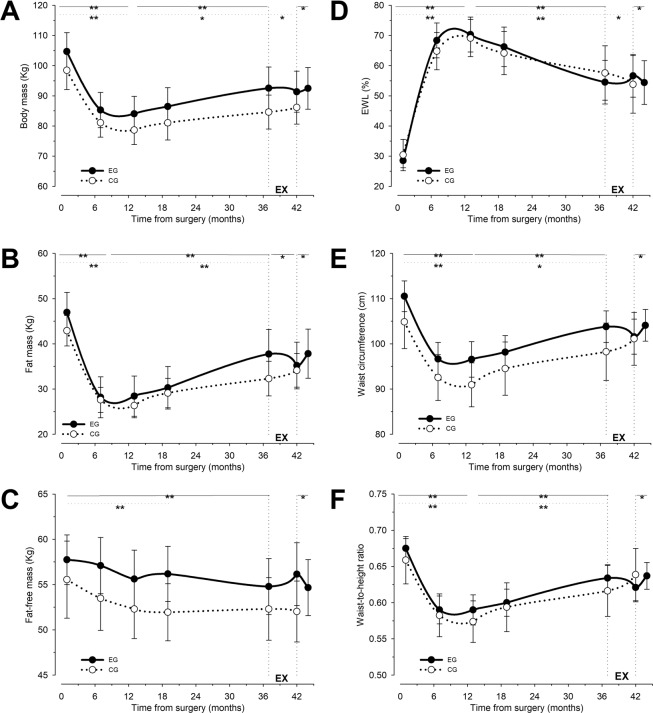
Changes in body mass (**A**), fat mass (**B**), fat-free mass (**C**), excess weight loss (**D**), waist circumference (**E**) and waist to height ratio (**F**) during the follow-up. The vertical dotted lines at 37 and 42 weeks mark the 5-month exercise intervention (EX). Data represent mean ± SEM. Data for the experimental group (EG) are indicated as black dots and continuous lines. Data for the control group (CG) are indicated as open dots and dotted lines. Statistical comparisons correspond to intragroup t-tests among the indicated time points. Horizontal continuous lines indicate the time points compared in the EG, while horizontal dotted lines indicate the time points compared in the CG. *p < 0.05; **p < 0.01. At the beginning of the intervention (EX) body mass (**A**) was significantly higher than after surgery, and this was due to the regain of fat mass (**B**) and a trend to lose fat-free mas (**C**). Consequently, at the beginning of the intervention a significant reduction of excess weight loss (**D**) was observed in both groups in comparison with its maximal value, reached approximately one year after surgery. This was accompanied by an increase of various cardiovascular risk factor (**E,F**). In the EG the intervention contributed to counteract the progressive increase in fat mass (**B**) and the decline in EWL (**D**) seen in the CG. Body mass was not significantly reduced during the intervention in the EG (**A**), possibly due to the trend to increasing the fat-free mass in this group caused by the intervention (**C**). Similarly, the intervention tended to improve the cardiovascular risk factors in the EG (**E,F**). All of these effects seem to be caused directly by the exercise program, since they significantly varied in the opposite direction in the retention evaluation, two months after ending the intervention.

The EG obtained its maximum decrease of total fat mass and body fat percentage 7 months after BS, whereas the CG did so 13 months after surgery. Both groups reached their maximum reduction of total weight and their maximum increase of %EWL by 13 months after surgery. Compared to that moment, weight loss, total fat mass (FM), and fat mass percentage (%FM), significant increased by 37 months after BS in both groups, in addition to a significant decrease of %EWL. This was paralleled by a significant reduction of fat free mass (FFM) at 37 months after surgery for the EG, and 19 months after BS for the CG (Fig. 2).

Changes in body composition and anthropometric measures after the exercise program are summarized in Table 2. After the intervention, the EG had significant reductions in total fat mass and fat mass percentage, showing a tendency to reduce the total weight and waist circumference, and to increase %EWL and FFM (Fig. 2). In contrast, the CG had a significant increase in total weight, fat mass and fat mass percentage, and significant reduction in %EWL. Before and after the intervention (37–42 months after BS), significant differences were observed between groups in the changes of all body composition and anthropometric measures except in hip circumference and FFM. However, FFM showed a large change between groups (*d* = 0.96). Forty-two months after surgery, in the CG there were no significant differences in the fat mass percentage when compared with one month after BS, nor differences in the FFM of the EG compared to one month after surgery (Supplementary material).

**Table 2:** Changes in body composition and anthropometric data after exercise program.

	Group	Start intervention^a^	Finish intervention^b^	Intra-group differences	Absolute Change	Inter-group differences
				(p-value)		(*p* value)
Weight (kg)	**EG**	92.5 ± 22.2	91.3 ± 21.5	0.195	−1.2 ± 2.6 (−3.07, 0.72)	**0.018**
	**CG**	84.6 ± 15.9	86.1 ± 15.6	**0.015**	1.5 ± 1.3 (0.41, 2.66)	
BMI (kg·m^−^²)	**EG**	34.4 ± 7.0	33.9 ± 6.7	0.191	−0.45 ± 1.0 (−1.18, 0.27)	**0.017**
	**CG**	32.8 ± 4.3	33.4 ± 4.3	**0.017**	0.62 ± 0.56 (0.15, 1.09)	
EWL (%)	**EG**	54.5 ± 22.8	56.6 ± 22.4	0.211	2.1 ± 5.0 (−1.43, 5.66)	**0.013**
	**CG**	57.6 ± 25.6	53.8 ± 27.0	**0.022**	− 3.8 ± 3.6 (−6.82, 0.72)	
FM (kg)	**EG**	37.7 ± 17.1	35.2 ± 16.3	**0.013**	−2.5 ± 2.6 (−4.41, 0.66)	**0.000**
	**CG**	32.3 ± 10.8	34.1 ± 10.5	**0.001**	1.8 ± 0.9 (1.01, 2.61)	
FM (%)	**EG**	39.4 ± 9.3	37.2 ± 9.8	**0.005**	−2.1 ± 1.8 (−3.45, 0.81)	**0.013**
	**CG**	37.5 ± 8.8	39.0 ± 8.05	**0.010**	1.5 ± 1.23 (0.49, 2.55)	
FFM (kg)	**EG**	54.8 ± 9.7	56.1 ± 11.0	0.072	1.4 ± 2.1 (−0.14, 2.88)	0.069
	**CG**	52.3 ± 9.8	52.0 ± 9.5	0.538	−0.3 ± 1.2 (−1.29, 0.74)	
FFM (%)	**EG**	60.6 ± 9.3	62.8 ± 9.8	**0.005**	2.1 ± 1.8 (0.82, 3.46)	**0.000**
	**CG**	62.5 ± 8.8	61.0 ± 8.0	**0.010**	−1.5 ± 1.2 (−2.55, −0.49)	
Waist (cm)	**EG**	103.6 ± 11.0	101.6 ± 12.2	0.227	−1.9 ± 4.9 (−5.49, 1.49)	**0.035**
	**CG**	98.2 ± 17.9	101.1 ± 16.6	0.072	2.8 ± 3.8 (−0.34, 6.1)	
Hip (cm)	**EG**	116.1 ± 19.2	116.0 ± 18.4	0.864	−0.152 (−2.09, 1.79)	0.305
	**CG**	113.3 ± 11.3	114.3 ± 11.2	0.126	1.01 (−0.36, 2.38)	
WtHR	**EG**	0.634 ± 0.06	0.621 ± 0.062	0.223	−0.013 ± 0.03 (−0.03, −0.001)	**0.033**
	**CG**	0.617 ± 0.101	0.637 ± 0.101	0.077	0.019 ± 0.26 (−0.003, 0.041)	

Data are mean ± SD. 95% confidence intervals in brackets. BMI, body mass index; EWL, excess weight loss; FM, fat mass; FFM, fat free mass; WtHR, waist-to-height ratio; EG, exercise group; CG, control group. Note: ^a^37 months after surgery; ^b^42 months after surgery.

Two months after the exercise program -i.e., between 42 and 44 months after BS-, the EG returned to exhibit weight regain, with increased fat mass and fat mass percentage, as well as decreased %EWL and FFM (Fig. 2).

### Cardio-metabolic risk factors and cardiorespiratory fitness

After the exercise program, the EG showed a significant reduction in blood glucose (75.9 ± 29.4 *vs*. 62.5 ± 13.6  mg·dL^−1^; *P* = 0.005, 95% CI: −19.6, −7.1) and total cholesterol (212.0 ± 49.9 *vs*. 187.4 ± 39.4  mg·dL^−1^; *P* = 0.026, 95% CI: −45.4, −3.8). In addition, the EG showed a small tendency to decrease systolic (132.5 ± 23.4 *vs*. 127.0 ± 20.2  mmHg; *d* = 0.25, 95% CI: −26.11, 15.05) and diastolic blood pressure (78.2 ± 9.3 *vs*. 75.1 ± 9.9  mmHg; *d* = 0.25, 95% CI: −14.9, 8.7), as well as CVR (1.7 ± 0.95 *vs*. 1.4 ± 0.70; *d* = 0.36, 95% CI: −1.08, 0.48). Between 37 months and 42 months, waist-to-height ratio had significant difference between groups (*P* = 0.033, 95% CI: −0.61, −0.01) (Table 2). Two months after the end of the exercise program (44 months after surgery), total cholesterol increased to 207.40 ± 51.0  mg·dL^−1^ (*P* = 0.019, 95% CI: 4.2, 35.8) and blood glucose to 70.7 ± 10.4  mg·dL^−1^ (*P* = 0.047, 95% CI: −3.2, 19.6) in the EG. Furthermore, at 44 months after BS increases in waist-to-height ratio (*P* = 0.004, 95% CI: 0.01, 0.02), CVR (*d* = 0.37, 95% CI: −0.38, 0.82), and diastolic blood pressure (*d* = 0.31, 95% CI: −7.6, 14.5) were observed in the EG.

Positive correlations were also observed between reductions in total weight and fat mass with reductions in cholesterol (*r* = 0.744, *P* = 0.014; *r* = 0.719, *P* = 0.019, respectively) and CVR (*r* = 0.808, *P* = 0.005; *r* = 0.679, *P* = 0.031, respectively), as well as between reductions in total weight and reductions in systolic blood pressure (*r* = 0.844, *P* = 0.002), after the intervention.

After the exercise program (42 months after surgery), the EG obtained significant improvements of absolute (1.7 ± 0.48 *vs*. 2.0 ± 0.67  l∙min^−1^; *P* = 0.005, 95% CI: 0.11, 0.47) and relative VO_2peak_ (19.8 ± 3.4 *vs*. 23.2 ± 4.6  ml∙kg^−1^ ∙ min^−1^; *P* = 0.002, 95% CI: 1.66, 5.1).

### Quality of life

After the intervention, the absolute change in bodily pain increased in the EG in comparison with the CG (Table 3). Additionally, in the EG changes in most of the components of the SF-36 showed medium to large effects sizes after the exercise program: role physical (*d* = 0.33, 95% CI: −35.2, 66.5), social functioning (*d* = 0.40, 95% CI: −15.8, 34.6), mental health (*d* = 0.63, 95% CI: −8.77, 33.8), vitality (*d* = 0.69, 95% CI: −7.69, 35.2), physical component summary (*d* = 0.55, 95% CI: −5.6, 17.9), and bodily pain (*d* = 1.04, 95% CI: −0.8, 54.5). In contrast, in the CG, there were no changes in most scales of the questionnaire. Only a decrease in the mental component summary (*d* = 0.72, 95% CI: −21.63, 4.26) and a small reduction in social functioning (*d* = 0.30, 95% CI: −49.0, 28.0) were found.

**Table 3:** Changes in SF-36 scores after exercise program.

	Group	Start intervention^a^	Finish intervention^b^	Intra-group differences (p-value)	Absolute Change	Inter-group differences
						(*p* value)
Physical functioning	**EG**	82.5 ± 21.7	86.9 ± 16.9	0.087	4.4 ± 6.2 (−0.83, 9.58)	0.121
	**CG**	88.7 ± 12.2	86.2 ± 19.8	0.502	−2.5 ± 10.0 (−10.9, 5.86)	
Role physical	**EG**	53.1 ± 50.8	68.7 ± 43.8	0.435	15.6 ± 53.3 (−29.0, 60.2)	0.276
	**CG**	71.9 ± 41.0	65.6 ± 42.1	0.170	−6.2 ± 11.6 (−15.9, 3.42	
Bodily pain	**EG**	44.5 ± 27.7	71.4 ± 23.7	0.089	26.9 ± 38.5 (−5.33, 59.1)	**0.044**
	**CG**	61.4 ± 33.4	57.0 ± 34.7	0.268	− 4.4 ± 10.3 (−13.0, 4.22)	
General health perceptions	**EG**	78.4 ± 18.7	82.5 ± 16.1	0.397	4.1 ± 13.0 (−6.68, 14,9)	0.454
	**CG**	71.9 ± 17.5	72.2 ± 20.2	0.830	0.37 ± 4.7 (−3.59, 4.34)	
Vitality	**EG**	65.6 ± 25.1	79.4 ± 12.9	0.118	13.7 ± 21.8 (−4.5, 32.0)	0.201
	**CG**	60.0 ± 18.9	61.9 ± 19.6	0.678	1.8 ± 12.3 (−8.34, 12.1)	
Social functioning	**EG**	78.1 ± 29.7	87.5 ± 14.9	0.442	9.3 ± 32.6 (−17.8, 36.6)	0.146
	**CG**	76.6 ± 34.3	65.6 ± 38.2	0.133	−10.9 ± 18.2 (−26.2, 4.3)	
Role emotional	**EG**	79.2 ± 39.6	75.0 ± 46.3	0.836	−4.2 ± 54.7 (−49.9, 41.6)	0.879
	**CG**	50.0 ± 47.1	41.7 ± 49.6	0.668	−8.3 ± 52.7 (−52.4, 35.7)	
Mental health	**EG**	75.0 ± 25.6	87.5 ± 11.4	0.250	12.5 ± 28.2 (−11.0, 36.0)	0.324
	**CG**	67.0 ± 22.0	68.5 ± 23.7	0.723	1.5 ± 11.5 (−8.11, 11.1)	
Physical component summary	**EG**	44.2 ± 9.9	50.0 ± 11.3	0.152	5.8 ± 0.10.2 (−2.74, 14.3)	0.165
	**CG**	49.5 ± 8.1	50.0 ± 14.1	0.842	.57 ± 7.8 (−5.95, 7.1)	
Mental component summary	**EG**	50.0 ± 13.9	51.6 ± 8.6	0.735	1.6 ± 12.5 (−8.91, 12.0)	0.270
	**CG**	47.5 ± 8.0	39.3 ± 15.1	0.153	−8.7 ± 15.3 (−21.5, 4.13)	

Data are mean ± SD. 95% confidence intervals in brackets. EG, exercise group; CG, control group. Note: ^a^37 months after surgery; ^b^42 months after surgery.

## Discussion

The effectiveness of BS on weight loss has been widely demonstrated. However, its effects are limited in time, and weight regain is a common complication in bariatric patients, mainly after restrictive procedures. In the literature, a dramatic increase in %EWL in the first months after surgery as well as a reduction of the FFM have been described^17^. Afterwards, weight loss slows down, until a point of maximum weight loss is reached between 12 and 24 months after surgery^3,5^. Nevertheless, decreases of %EWL from 56% to 46% have been reported in subjects submitted to sleeve gastrectomy between 12 and 36 months after surgery^18^. In the same way, other studies have observed significant weight regain between 24 and 36 months^19^, and between 24 and 48 months after surgery^3^. Our data are in accordance with these previous observations. In our sample, the large increase in %EWL during the first months was followed by a small increase until month 13 after surgery, when the highest %EWL was reached. At this time-point, a reduction of %EWL began, causing a significant weight regain in both groups at 37 months after surgery.

Unfortunately, there are few studies in the literature in which body composition has been measured in the long-term. To our knowledge, most studies have measured body composition between 6 and 24 months after surgery. According to these studies, a biphasic loss of fat mass can be observed: an initial fast phase of fat mass reduction during the first year after surgery, that is followed by a second phase in which fat loss slows down until it reaches a point of maximum fat mass loss at month 24^20^. However, studies that have a longer follow-up period showed a tendency to recover fat mass^21,22^. Similar to these reports, in the present study, patients reached their maximum fat loss between month 7 and 13 months after surgery. From this moment onwards, an increase in fat mass was observed in both groups, being significantly higher 37 months after surgery. Additionally, significant losses of FFM were observed in both groups.

Several mechanisms may contribute to the weight regain after BS. On the one hand, the decrease of leptin and insulin levels may, in turn, generate a greater appetite sensation^23^. In this regard, a progressive increase in energy intake^24^ has been reported in BS patients over time. Poor eating habits^12^ as well as an increase of the gastric cavity^11^ may contribute to this effect after sleeve gastrectomy. On the other hand, weight loss also leads to a reduction of basal metabolic rate^25^, which may be an important component after BS, since associations have been found between low basal metabolic rate and weight regain^26^.

According to the results of the present study, individualized and supervised exercise programs may be considered as adjuvant interventions to prevent weight regain in the long-term after sleeve gastrectomy. Our data showed significant reductions in fat mass and fat mass percentage, a trend towards total weight reduction and an increase of %EWL and FFM in the EG, at the end of the exercise program. However, during the corresponding time after surgery, in the CG weight, fat mass and fat mass percentage continued to increase significantly, while significant reductions of %EWL occurred. This resulted in fat mass reaching similar values to the preoperative ones in the CG at 42 months after surgery.

Unfortunately, studies found in the literature on this topic are scarce and mostly observational. These studies have shown positive associations between low levels of self-reported physical activity^13^, a sedentary lifestyle^4^ and sitting time with weight regain^27^, as well as that subjects who performed one or more weekly sessions of physical activity had a lower weight regain^27^. To the best of our knowledge, only an experimental study performed a physical exercise program in the medium-term after BS. This study was conducted in patients with a BMI greater than 30  kg·m^−2^ 12–24 months after surgery, reporting weight and fat mass reductions only in the exercise group, while in the control group these variables increased^28^. However, in contrast with our data, a trend to decrease FFM was observed. This difference may be explained by the introduction of resistance training of controlled intensity (≥70% RM) in our intervention. This shows the effectiveness and necessity of resistance training at high intensities in bariatric patients, since an increase in FFM leads to an increase in the basal metabolic rate, which can help to prevent weight regain.

Two months after the end of the exercise program, significant increases in total weight and fat mass and significant reductions of %EWL and FFM were observed in the EG. Therefore, the benefits of the exercise program were not retained after its ending. Weight recovery after a program of weight loss has been previously related to the reduction of physical activity in patients with obesity^29^. In fact, it has been concluded that physical activity is a determining factor in the maintenance of weight loss^30^. Our data support this argument.

Sleeve gastrectomy induces a reduction in CVR factors^2^. Although these reductions tend to persist in the long-term, there is a proportion of patients in which improvements in comorbidities tend to be attenuated^10^. For example, relapse in type 2 diabetes mellitus is a common complication, with relapse rates ranging from 19% to 35.1%^31,32^. In addition, type 2 diabetes mellitus relapses have been associated with weight regain^32,33^. Our results show that after the exercise program, both blood glucose and cholesterol had significantly reduced. In the case of cholesterol, reductions were also relevant from a clinical point of view as cholesterol levels ranged into a desirable zone (<200  mg·dl^−1^)^34^ after the intervention.

Exercise contributes to the observed CVR factors reductions. Both endurance and resistance training trigger a large uptake of glucose in the skeletal muscle^35^. High intensity interval training (HIIT) seems to be of great importance, since it causes a higher glucose uptake than moderate intensity training^36^. This can be explained by different mechanisms. First, HIIT increases the sarcolemmal translocation of the glucose transporter GLUT4^37^. Additionally, HIIT increases skeletal muscle oxidative capacity^38^, which is impaired in subjects with obesity^39^. This may increase insulin sensitivity, since there is an association between both^40^. Therefore, HIIT may contribute to a reduction in hyperglycaemia^38^ and insulin resistance^41^. In addition, the exercise program provoked reductions in waist circumference and waist-to-hip ratio. Taken together, all these effects are of great importance, since they are associated with cardio-metabolic risk^42^.

Along with these physiological responses, improvements were observed in the quality of life of participants who performed the exercise program, especially in the physical scales of the SF36 questionnaire. Many studies have reported short-term improvements in quality of life after BS, both in the physical and mental scales^43^. However, these effects are lost in the long-term, especially in the physical domains^44,45^. The results of the present study show that exercise can reverse this trend, improving the participant’s quality of life, particularly in the physical components.

Additionally, other studies have shown that between the 1^st^ and 3^rd^ year after surgery bodily pain increases in BS patients^44^. Our data have shown a reduction of bodily pain in the participants in the exercise program. This result can be due to the addition of two factors: weight loss and resistance training, which generates a lower mechanical load and provides greater support and joint stability^46^.

Nevertheless, several limitations of the present study should be recognized. First, this study had a lack of nutritional and physical activity control, and participants were only encouraged to follow the guidelines established by their hospital. Therefore, it is possible that subjects who lost weight, in addition to performing exercise would have changed their eating habits. Consequently, future works should test if these variables are affected by an exercise-based intervention like the one described here. Additionally, the small sample size may make it difficult to generalize our results.

In summary, our data show that a point of maximum weight loss and fat mass is reached 13 months after sleeve gastrectomy. From this moment on, a process of weight regain begins, until there is a significant recovery of weight and fat mass at 37 months after the surgery. An individualized and supervised combined exercise program as described in this study slows down this weight regain and generates a reduction of fat mass and a tendency to increase fat-free mass. Moreover, improvements in cardiorespiratory fitness, cardio-metabolic risk factors and in health-related quality of life are observed after the exercise program. These improvements disappear two months after the end of the exercise program. Therefore, our results show that exercise is an effective tool to avoid weight regain and relapse in cardio-metabolic risk even at long time after sleeve gastrectomy. Consequently, implementation of exercise programs in post bariatric patients should be emphazised.

## Methods

### Participants

A prospective randomized clinical trial (ClinicalTrials.gov number: NCT03603392; 04/07/2018) was performed. A total of 40 patients who underwent sleeve gastrectomy during three consecutive years in two university hospitals from an urban area (≈230000 inhabitants), were offered to join the study. Patients with previous cardiovascular diseases, history of cancer in the previous 5 years and functional limitations that prevented them from performing the exercise program were excluded from the study. All the patients signed an informed consent form to be included in the study. This study was conformed to the Declaration of Helsinki and approved by the University Miguel Hernández Ethics Committee. Group sizes were estimated to provide a statistical power of 0.80 with a significance level of 0.01, according to our previous experience with this population and intervention^47^.

### Design

Body composition and anthropometric measurements were assessed in all participants at 5 time-points (1, 7, 13, 19 and 37 months after surgery) before starting the intervention. At 37 months after BS, participants were randomized in two groups: an exercise group (EG; n = 11 patients coming from the twenty first patients that were operated on) and a control group (CG; n = 10 patients, coming from the resting twenty patients). Patients form both hospitals were included in each group. The EG performed a 5-months of supervised exercise program, while the CG followed the usual care. Measurements of cardiorespiratory fitness, biochemical parameters, body composition, anthropometry, blood pressure and health-related quality of life for the EG were performed. Body composition, anthropometry and health-related quality of life for the CG were also assessed. Data were collected at the start of the exercise program (37 months after BS) and at the end of the program (42 months after BS). Finally, the EG was evaluated 2 months after the end of the exercise program (44 months after BS).

Measurements were performed in a climatically controlled laboratory, located at 80  m above sea level, in which the relative air humidity was maintained between 45–60% and the temperature between 22–24 °C.

### Exercise program

The exercise program lasted 20 weeks, which were distributed in five 4-week blocks (Table 4). All training sessions were performed in the sport facilities of our institution and supervised by graduates in sports sciences. The first block included two weekly sessions, in which endurance and resistance training for 5 muscle groups (hamstrings, pectorals, quadriceps, latissimus dorsi, and gastrocnemius) was combined in the same session. In the second block participants trained 3 days a week: in two of them a high-intensity interval training (HIIT) was performed, followed by resistance training (biceps and triceps were added to the previous 5 major muscle groups trained), while in the third session only the endurance training was performed. Between the third and fifth block participants trained 4 days per week. In two sessions, a HIIT was performed, followed by resistance training, focusing on different muscle groups in each session: (a) session 1: pectorals, quadriceps, biceps, and hamstrings; (b) session 2: dorsal, triceps, gastrocnemius and deltoids. In the other two sessions, endurance training was performed. In all the blocks, 2 days a week of flexibility training were carried out.

The endurance training (performed either on cycle-ergometer, elliptical or treadmill) was monitored using a heart rate monitor (FT40, Polar, Finland). The resistance training was performed using resistance machines, and training intensity was determined by percentages of 1 maximum repetition which was estimated using the Brzycki formula^48^. The HIIT consisted of a five-minute warm up, increasing the intensity from 40% of VO_2peak_ to 60% VO_2peak_. After this, 20  minutes with bouts of 30  seconds at high intensity (60–95% VO_2peak_) and 30  seconds of active recovery (40% VO_2peak_), for a total of 10  minutes of training at high intensity were performed. Finally, 3  minutes of cool-down at 40% VO_2peak_ were performed. During the two last weeks of block 3, both the RM and the VO_2peak_ were measured, and the intensities of the following sessions (blocks 4 and 5) were fixed according to these values.

Resistance training, HIIT and flexibility exercises were combined in the same session, in order to reach similar duration per session (roughly 50  min) to the continuous aerobic training.

**Table 4:** Exercise program performed by the experimental group.

		Block 1	Block 2	Block 3	Block 4	Block 5
**Sessions per week**		2	3	4	4	4
**Resistance training**		2 d/w	2 d/w	2 d/w	2 d/w	2 d/w
		1 set	1 set	4 sets	4 sets	4 sets
		5 exercises	7 exercises	4 exercises	4 exercises	4 exercises
		20 repetitions	20 repetitions	15 repetitions	12 repetitions	10 repetitions
		~50% RM	~60% RM	~65% RM	~70% RM	~75% RM
		8 min	12 min	28 min	26 min	24 min
**Endurance training**	**HIIT**	—	2 d/w	2 d/w	2 d/w	2 d/w
			60–70% VO_2peak_	70–80% VO_2peak_	75–85% VO_2peak_	80–95% VO_2peak_
			20 min	20 min	20 min	20 min
	**ACT**	2 d/w	1 d/w	2 d/w	2 d/w	2 d/w
		60–70% HR_max_	70–75% HR_max_	70–80% HR_max_	70–80% HR_max_	70–80% HR_max_
		35 min	50 min	50 min	50 min	50 min
**Stretching training**		2 d/w	2 d/w	2 d/w	2 d/w	2 d/w
		4 exercises	4 exercises	4 exercises	4 exercises	4 exercises
		1 min per exercise	1 min per exercise	1 min per exercise	1 min per exercise	1 min per exercise

d/w indicates days per week; RM, repetition maximum; ACT, aerobic continuous training; HR_max_, maximal heart rate; HIIT, high intensity interval training; VO_2peak_, peak oxygen uptake. Blocks 1 and 2 were considered as a progressive adaptation. During the second half of block 3, the participants were evaluated and the new values of RM and VO_2peak_ were used to prescribe the exercise intensity from that moment on. HIIT, resistance training and stretching were combined in the same session. Therefore, independently of its modality (concurrent vs aerobic continuous exercise), every session had a similar duration (≈50  min).

### Test measures

#### Body composition and anthropometric measures

Body composition and anthropometric measures were evaluated between 8:00–9:00 AM, after at least 10  hours of fasting. Alcohol consumption and physical activity were restricted for at least 8  hours before the test^49^ while physical activity was forbidden in the 48  hours prior to the test. Participants performed the assessment with an empty bladder^49^. Bioimpedance analysis (Tanita, TBF 300 A, Tokyo, Japan) was used to measure body weight and body composition. BMI was calculated and expressed as kg·m^−2^ ^50^. The percentage of EWL was calculated according with the usual formula: %EWL = (initial weight − current weight)/(actual weight −(25 × height²)) × 100)^51^. The International Society for the Advancement of Kinanthropometry (ISAK) protocol^52^ was used to assess height as well as waist and hip circumferences.

#### Cardiorespiratory fitness

Cardiorespiratory fitness was evaluated between 4:00–8:00 PM. The peak oxygen uptake (VO_2peak_) was determined on a cycle ergometer (Technogym Bike Med, Technogym, Gambettola, Italy), using an Oxycon Pro gas analysis system (Jaeger, Friedberg, Germany). For testing, the protocol described by Achten *et al*.^53^ was adapted. The protocol had two phases: (a) a 4-minute warm-up at 40 watts, followed by increases of 20 watts every 3  minutes, maintaining a cadence of 60 rotations per minute until the respiratory exchange ratio reached 1.0; and, (b) a second phase that started at this moment, and that consisted of increments of 20 watts every minute, maintaining a cadence between 70–80 rotations per minute, until volitional fatigue. The VO_2peak_ was calculated as the average of the highest 30  seconds of oxygen uptake. The VO_2peak_ was expressed in absolute values (l∙min^−1^) and normalized to the total body weight (ml∙kg^−1^ ∙ min^−1^).

#### Cardio-metabolic risk factors

Cardio-metabolic risk factors were measured between 8:00–9:00 AM, after at least 10  hours of fasting. Exercise was forbidden in the 48  hours prior to the test. Blood pressure was measured using a digital sphygmomanometer (Microlife WatchBP Home, Heerbrugg, Switzerland), according to established recommendations^54^. Capillary blood samples were taken to analyze blood glucose and total cholesterol using a portable multi-analyzer (Accutrend GCT, Roche Diagnostics, Mannheim, Germany).

#### Cardiovascular risk

The SCORE risk charts of the European Society of Cardiology^55^ were used to estimate the 10-year coronary risk. The formula uses total cholesterol, systolic blood pressure, gender, age, and smoking status^55^. In addition, cardiovascular risk was also estimated using waist-to-height ratio^56^.

#### Health-related quality of life

The health-related quality of life was measured by the Short Form Health Survey 36 (SF-36)^57^, in its version adapted to the Spanish context^58^. This questionnaire measures physical and mental health through 8 scales, the scores of which are transformed to values between 0–100 points, with the highest scores meaning a better function. These 8 scales are grouped into two summary components, the physical summary component, and the mental summary component^59^, which were calculated according to the reference values of the Spanish population^60^, with a mean of 50 and a standard deviation of 10.

### Statistical analysis

The statistical package SPSS 22.0 (SPSS Inc., Chicago, IL, USA) was used to analyze the data. Graphs were drawn with Sigmaplot v.11.0 (Systat Software, Inc.). The Kolmogorov-Smirnov test was used to determine if the data were normally distributed. A one-way repeated measurements ANOVA was used to evaluate the influence of time on body composition and CVR factors intragroup. In addition, the paired T-test was used to analyze the intra-group changes in health-related quality of life and in physical fitness during exercise program. In the cases in which the variable did not show a normal distribution, a Wilcoxon paired test was used. For the inter-group comparisons, the one-way ANOVA was used. The associations between body composition and CVR factors were performed using Pearson’s bivariate correlation analysis. To calculate the Effect Size, the Cohen’s *d* was used and was interpreted as follows: 0.20–0.50 (small), 0.50–0.80 (medium), >0.80 (large)^61^. Significant differences were considered when *p* < 0.05. Unless otherwise indicated, data are presented as mean ± SD.

Blood pressure and CVR of one participant of the EG were eliminated in the last measurement because she began to take antihypertensive medication.

## Supplementary information

Supplementary table 1.

## Data Availability

All data are available from the corresponding author (M-R) upon reasonable request.

## References

[1] Reinhold RB (1982). Critical analysis of long term weight loss following gastric bypass. Surg. Gynecol. Obstet..

[2] Ricci C, Gaeta M, Rausa E, Macchitella Y, Bonavina L (2014). Early impact of bariatric surgery on type II diabetes, hypertension, and hyperlipidemia: a systematic review, meta-analysis and meta-regression on 6,587 patients. Obes. Surg..

[3] Magro DO (2008). Long-term weight regain after gastric bypass: A 5-year prospective study. Obes. Surg..

[4] Freire RH, Borges MC, Alvarez-Leite JI, Toulson Davisson Correia MI (2012). Food quality, physical activity, and nutritional follow-up as determinant of weight regain after Roux-en-Y gastric bypass. Nutrition.

[5] Felsenreich DM (2016). Weight loss, weight regain, and conversions to Roux-en-Y gastric bypass: 10-year results of laparoscopic sleeve gastrectomy. Surg. Obes. Relat. Dis..

[6] Bohdjalian A (2010). Sleeve gastrectomy as sole and definitive bariatric procedure: 5-Year results for weight loss and ghrelin. Obes. Surg..

[7] Lemanu DP (2015). Five-year results after laparoscopic sleeve gastrectomy: A prospective study. Surg. Obes. Relat. Dis..

[8] Himpens J, Dobbeleir J, Peeters G (2010). Long-term results of laparoscopic sleeve gastrectomy for obesity. Ann. Surg..

[9] Alexandrou A (2015). Laparoscopic sleeve gastrectomy for morbid obesity: 5-year results. Am. J. Surg..

[10] Shah M, Simha V, Garg A (2006). Review: Long-term impact of bariatric surgery on body weight, comorbidities, and nutritional status. J. Clin. Endocrinol. Metab..

[11] Alvarez V (2016). Mechanisms of long-term weight regain in patients undergoing sleeve gastrectomy. Nutrition.

[12] Odom J (2010). Behavioral Predictors of Weight Regain after Bariatric Surgery. Obes. Surg..

[13] Livhits M (2011). Patient behaviors associated with weight regain after laparoscopic gastric bypass. Obes. Res. Clin. Pract..

[14] Herring LY (2016). Changes in physical activity behaviour and physical function after bariatric surgery: A systematic review and meta-analysis. Obes. Rev..

[15] Josbeno DA, Kalarchian M, Sparto PJ, Otto AD, Jakicic JM (2011). Physical Activity and Physical Function in Individuals Post-bariatric Surgery. Obes Surg.

[16] Kennedy L (2006). Lifestyle, Diabetes, and Cardiovascular Risk Factors 10 Years After Bariatric. Surgery. Yearb. Med..

[17] Otto M (2015). Sleeve Gastrectomy and Roux-en-Y Gastric Bypass Lead to Comparable Changes in Body Composition after Adjustment for Initial Body Mass Index. Obes. Surg..

[18] Coleman KJ (2014). Three-year weight outcomes from a bariatric surgery registry in a large integrated healthcare system. Surg. Obes. Relat. Dis..

[19] Blume CA (2012). Nutritional profile of patients before and after Roux-en-Y gastric bypass: 3-year follow-up. Obes. Surg..

[20] Biagioni MFG (2017). Bariatric Roux-En-Y Gastric Bypass Surgery: Adipocyte Proteins Involved in Increased Bone Remodeling in Humans. Obes. Surg..

[21] Lubrano C (2004). Reduction of risk factors for cardiovascular diseases in morbid-obese patients following biliary-intestinal bypass: 3 years’ follow-up. Int. J. Obes. Relat. Metab. Disord..

[22] Laguna S (2016). Las variaciones en colesterol-HDL tras bypass gástrico proximal son independientes de la evolución ponderal. An. Sist. Sanit. Navar..

[23] Sumithran P, Proietto J (2013). The defence of body weight: a physiological basis for weight regain after weight loss. Clin Sci (Lond)..

[24] Sjöström L (2004). Lifestyle, Diabetes, and Cardiovascular Risk Factors 10 Years after Bariatric Surgery. N. Engl. J. Med..

[25] Browning MG, Franco RL, Cyrus JC, Celi F, Evans RK (2016). Changes in Resting Energy Expenditure in Relation to Body Weight and Composition Following Gastric Restriction: A Systematic Review. Obes. Surg..

[26] Faria SL, Kelly E, Faria OP (2009). Energy Expenditure and Weight Regain in Patients Submitted to Roux-en-Y Gastric Bypass. Obes. Surg..

[27] Herman KM, Carver TE, Christou NV, Andersen RE (2014). Keeping the weight off: Physical activity, sitting time, and weight loss maintenance in bariatric surgery patients 2 to 16 years postsurgery. Obes. Surg..

[28] Herring LY (2017). The effects of supervised exercise training 12 – 24 months after bariatric surgery on physical function and body composition: a randomised controlled trial. Int J Obes.

[29] Wang X (2009). Weight Regain is Related to Decreases in Physical Activity During Weight Loss. Med Sci Sport. Exerc.

[30] Anastasiou CA, Karfopoulou E, Yannakoulia M (2015). Weight regaining: From statistics and behaviors to physiology and metabolism. Metabolism: Clinical and Experimental.

[31] Arterburn DE (2013). A multisite study of long-term remission and relapse of type 2 diabetes mellitus following gastric bypass. Obes. Surg..

[32] Brethauer Stacy A., Aminian Ali, Romero-Talamás Héctor, Batayyah Esam, Mackey Jennifer, Kennedy Laurence, Kashyap Sangeeta R., Kirwan John P., Rogula Tomasz, Kroh Matthew, Chand Bipan, Schauer Philip R. (2013). Can Diabetes Be Surgically Cured? Long-Term Metabolic Effects of Bariatric Surgery in Obese Patients with Type 2 Diabetes Mellitus. Annals of Surgery.

[33] Jimenez A (2012). Long-Term Effects of Sleeve Gastrectomy and Roux-en-Y Gastric Bypass Surgery on Type 2 Diabetes Mellitus in Morbidly Obese Subjects. Ann.Surg..

[34] National Cholesterol Education Program (2002). Third report of the National Cholesterol Education Program (NCEP) expert panel on detection, evaluation, and treatment of high blood cholesterol in adults. Circulation.

[35] Daugaard JR, Richter EA (2001). Relationship between muscle fibre composition, glucose transporter protein 4 and exercise training: possible consequences in non-insulin-dependent diabetes mellitus. Acta Physiol. Scand..

[36] Sylow L, Kleinert M, Richter EA, Jensen TE (2017). Exercise-stimulated glucose uptake - regulation and implications for glycaemic control. Nat. Rev. Endocrinol..

[37] Hood MS, Little JP, Taoplsky MA, Frank M, Gibala MJ (2011). Low-volume interval training improves muscle oxidative capacity in sedentary adults. Med Sci Sport. Exerc.

[38] Little JP (2011). Low-volume high-intensity interval training reduces hyperglycemia and increases muscle mitochondrial capacity in patients with type 2 diabetes. J. Appl. Physiol..

[39] Houmard JA, Pories WJ, Dohm GL (2013). Severe obesity: evidence for a deranged metabolic program in skeletal muscle?. Exerc Sport Sci Rev.

[40] Kelley DE (2005). Skeletal muscle fat oxidation: timing and flexibility are everything. J. Clin. Invest..

[41] Jelleyman C (2015). The effects of high-intensity interval training on glucose regulation and insulin resistance: A meta-analysis. Obes. Rev..

[42] Ashwell M, Gunn P, Gibson S (2012). Waist-to-height ratio is a better screening tool than waist circumference and BMI for adult cardiometabolic risk factors: Systematic review and meta-analysis. Obesity Reviews.

[43] Omotosho P, Mor A, Shantavasinkul PC, Corsino L, Torquati A (2016). Gastric bypass significantly improves quality of life in morbidly obese patients with type 2 diabetes. Surg. Endosc..

[44] King WC (2016). Change in Pain and Physical Function Following Bariatric Surgery for Severe Obesity. Jama.

[45] Strain GW (2011). Cross-sectional review of effects of laparoscopic sleeve gastrectomy at 1, 3, and 5 years. Surg. Obes. Relat. Dis..

[46] Zdziarski LA, G WJ, Vincent HK (2015). Chronic pain management in the obese patient: a focused review of key challenges and potential exercise solutions. J Pain Res..

[47] Marc-Hernández A., Ruiz-Tovar J., Aracil A., Guillén S., Moya-Ramón Manuel (2019). Impact of Exercise on Body Composition and Cardiometabolic Risk Factors in Patients Awaiting Bariatric Surgery. Obesity Surgery.

[48] Brzycki M (1993). Strength Testing—Predicting a One-Rep Max from Reps-to-Fatigue. J. Phys. Educ. Recreat. Danc..

[49] Kyle UG (2004). Bioelectrical impedance analysis - Part II: Utilization in clinical practice. Clin. Nutr..

[50] National Institutes of Health (1998). National Heart, Lung, and B. I. Clinical guidelines on the identification, evaluation, and treatment of overweight and obesity in adults. The Evidence Report. Obes. Res..

[51] Deitel M, Greenstein R (2003). Recommendations for Reporting Weight Loss. Obes. Surg..

[52] Stewart, A., Marfell-Jones, M., Olds, T. & Ridder, H. de. *International Standards for Anthropometric Assessment*. (International Society for the Advancement of Kinanthropometry, 2011).

[53] Achten J, Gleeson M, Jeukendrup AE (2002). Determination of the exercise intensity that elicits maximal fat oxidation. Med. Sci. Sports Exerc..

[54] Pickering TG (2005). Recommendations for Blood Pressure Measurement in Humans and Experimental Animals: Part 1: Blood Pressure Measurement in Humans: A Statement for Professionals From the Subcommittee of Professional and Public Education of the American Heart Association Cou. Hypertension.

[55] Piepoli MF (2016). 2016 European Guidelines on cardiovascular disease prevention in clinical practice. European Heart Journal.

[56] Félix-Redondo FJ (2013). Prevalence of obesity and associated cardiovascular risk: the DARIOS study. BMC Public Health.

[57] Ware, J. E., Snow, K. K., Kosinski, M. & Gandek, B. *SF-36 health survey: manual and interpretation guide*. (1993).

[58] Alonso J, Prieto L, Antó JM (1995). La versión española del SF-36 Health Survey (Cuestionario de Salud SF-36): un instrumento para la medida de los resultados clínicos. Med. Clin..

[59] Ware, J. E., Snow, K. K., Kosinski, M. & Gandek, B. *SF-36 physical and mental health summary scales: a user’s manual*. (Health Institute New England Medical Center, 1994).

[60] Alonso J (1998). Valores poblacionales de referencia de la versión Española del cuestionario de salud SF-36. Med. Clin. (Barc)..

[61] Cohen, J. *Statistical Power Analysis for the Behavioral Sciences*. (Routledge, 2013).

