# Supplementary material for: Effects of a High-Intensity Exercise Program on Weight Regain and Cardio-metabolic Profile after 3 Years of Bariatric Surgery: A Randomized Trial
Source: Sci Rep. 2020 Feb 20;10:3123. doi: 10.1038/s41598-020-60044-z (PMC7033151; doi:10.1038/s41598-020-60044-z)
Supplement: Supplementary file 1 — Supplementary table 1. [file 41598_2020_60044_MOESM1_ESM.docx]

| **Supplementary table 1.** Body composition and anthropometric data. | | | | | | | | |
| --- | --- | --- | --- | --- | --- | --- | --- | --- |
|  | | **1 month**  **after surgery** | **7 months**  **after surgery** | **13 months**  **after surgery** | **19 months**  **after surgery** | **37 months**  **after surgery** | **42 months**  **after surgery** | **44 months**  **after surgery** |
| **Weight (kg)** | **EG** | 104.7 ± 19.8 | 85.3 ± 18.6 ^a^ | 84.0 ± 18.2 ^a^ | 86.5 ± 19.8 ^a^ | 92.5 ± 22.2 ^a b c d^ | 91.3 ± 21.5 ^a b c d^ | 92.5 ± 21.9 ^a b c d f^ |
|  | **CG** | 98.4 ± 18.0 | 81.1 ± 13.5 ^a^ | 78.6 ± 13.5 ^a^ | 81.0 ± 16.1 ^a^ | 84.6 ± 15.9 ^a b c^ | 86.1 ± 15.6 ^a b c e^ | -- |
| **BMI (kg·m^-^²)** | **EG** | 38.9 ± 4.8 | 31.7 ± 5.3 ^a^ | 31.2 ± 5.4 ^a^ | 32.1 ± 6.0 ^a^ | 34.4 ± 7.0 ^a b c d^ | 33.9 ± 6.7 ^a b c d^ | 34.3 ± 6.8 ^a b c d f^ |
|  | **CG** | 38.2 ± 5.1 | 31.4 ± 3.1 ^a^ | 30.5 ± 3.2 ^a^ | 31.3 ± 3.7 ^a^ | 32.8 ± 4.3 ^a b c^ | 33.4 ± 4.3 ^a b c d e^ | -- |
| **FM (kg)** | **EG** | 46.9 ± 14.0 | 28.2 ± 14.3 ^a^ | 28.4 ± 14.1 ^a^ | 30.3 ± 14.9 ^a c^ | 37.7 ± 17.1 ^a b c d^ | 35.2 ± 16.3 ^a b c d e^ | 37.8 ± 17.2 ^a b c d f^ |
|  | **CG** | 42.9 ± 9.5 | 27.6 ± 7.9 ^a^ | 26.3 ± 7.6 ^a^ | 29.1 ± 9.2 ^a^ | 32.3 ± 10.8 ^a b c^ | 34.1 ± 10.5 ^a b c d e^ | -- |
| **FM (%)** | **EG** | 44.2 ± 6.2 | 31.7 ± 10.1 ^a^ | 32.6 ± 10.2 ^a^ | 33.6 ± 9.8 ^a^ | 39.4 ± 9.3 ^a b c d^ | 37.2 ± 9.8 ^a b c d e^ | 39.5 ± 9.5 ^a b c d f^ |
|  | **CG** | 43.5 ± 5.8 | 33.8 ± 7.3 ^a^ | 33.2 ± 6.9 ^a^ | 35.3 ± 6.6 ^a^ | 37.5 ± 8.8 ^a b c^ | 39.0 ± 8.05 ^b c d e^ | -- |
| **FFM (kg)** | **EG** | 57.7 ± 8.7 | 57.1 ± 9.8 | 55.6 ± 10.1 | 56.2 ± 9.6 ^a^ | 54.8 ± 9.7 ^a b d^ | 56.1 ± 11.0 | 54.7 ± 9.8 ^a b d f^ |
|  | **CG** | 55.5 ± 12.1 | 53.5 ± 9.9 | 52.3 ± 9.2 ^a^ | 51.9 ± 8.9 ^a^ | 52.3 ± 9.8 | 52.0 ± 9.5 | -- |
| **FFM (%)** | **EG** | 55.8 ± 6.2 | 68.2 ± 10.1 ^a^ | 67.4 ± 10.2 ^a^ | 66.4 ± 9.8 ^a^ | 60.6 ± 9.3 ^a b c d^ | 62.8 ± 9.8 ^a b c d e^ | 60.5 ± 9.5 ^a b c d f^ |
|  | **CG** | 56.5 ± 5.8 | 66.2 ± 7.3 ^a^ | 66.8 ± 6.9 ^a^ | 64.7 ± 6.6 ^a^ | 62.5 ± 8.8 ^a b c^ | 61.0 ± 8.0 ^a b c e^ | -- |
| **Waist (cm)** | **EG** | 110.5 ± 10.8 | 96.6 ± 11.6 ^a^ | 96.5 ± 12.4 ^a^ | 98.2 ± 11.5 ^a^ | 103.6 ± 11.0 ^a b c d^ | 101.6 ± 12.2 ^a c^ | 104.1 ± 11.1 ^a b c d f^ |
|  | **CG** | 104.8 ± 16.7 | 92.5 ± 14.3 ^a^ | 90.9 ± 13.7 ^a^ | 94.5 ± 16.7 ^a c^ | 98.2 ± 17.9 ^b c^ | 101.1 ± 16.6 ^b c d^ | -- |
| **Hip (cm)** | **EG** | 124.5 ± 13.7 | 111.1 ± 14.5 ^a^ | 109.6 ± 14.3 ^a^ | 112.2 ± 18.2 ^a^ | 116.1 ± 19.2 ^a c^ | 116.0 ± 18.4 ^a c d^ | 117.1 ± 18.9  ^a c d f^ |
|  | **CG** | 123.4 ± 10.7 | 108.0 ± 8.5 ^a^ | 106.7 ± 9.3 ^a^ | 111.2 ± 10.6 ^a c^ | 113.3 ± 11.3 ^a b c^ | 114.3 ± 11.2 ^a b c^ | -- |
| T, test; BMI, body mass index; FM, fat mass; FFM, fat-free mass.  ^a^ significant differences from 1 month after surgery (*p* < 0.05); ^b^ significant differences from 7 months after surgery (*p* < 0.05); ^c^ significant differences from 13 months after surgery (*p* < 0.05); ^d^ significant differences from 19 months after surgery (*p* < 0.05); ^e^ significant differences from 37 months after surgery (*p* < 0.05); ^f^ significant differences from 42 months after surgery (*p* < 0.05). | | | | | | | | |

**Effects of a High-Intensity Exercise Program on Weight Regain and Cardio-metabolic Profile after 3 Years of Bariatric Surgery: A Randomized Trial.**

**Marc-Hernández A, Ruiz-Tovar J, Aracil A, Guillén S, Moya-Ramón M.**
